# Exposure to *Batrachochytrium dendrobatidis* affects chemical defences in two anuran amphibians, *Rana dalmatina* and *Bufo bufo*

**DOI:** 10.1186/s12862-021-01867-w

**Published:** 2021-07-03

**Authors:** János Ujszegi, Krisztina Ludányi, Ágnes M. Móricz, Dániel Krüzselyi, László Drahos, Tamás Drexler, Márk Z. Németh, Judit Vörös, Trenton W. J. Garner, Attila Hettyey

**Affiliations:** 1 Lendület Evolutionary Ecology Research Group, Plant Protection Institute, Centre for Agricultural Research, Herman Ottó út 15, Budapest, 1022 Hungary; 2 Department of Pharmaceutics, Faculty of Pharmacy, Semmelweis University, Hőgyes Endre utca 7, Budapest, 1092 Hungary; 3 Department of Pathophysiology, Plant Protection Institute, Centre for Agricultural Research, Herman Ottó út 15, Budapest, 1022 Hungary; 4 MS Proteomics Research Group, Institute of Organic Chemistry, Research Centre for Natural Sciences, Magyar tudósok körútja 2, Budapest, 1117 Hungary; 5 Department of Ecology, Institute for Biology, University of Veterinary Medicine, Rottenbiller utca 50, Budapest, 1077 Hungary; 6 Department of Plant Pathology, Plant Protection Institute, Centre for Agricultural Research, Herman Ottó út 15, Budapest, 1022 Hungary; 7 Department of Zoology, Hungarian Natural History Museum, Baross street 13, Budapest, 1088 Hungary; 8 Institute of Zoology, Zoological Society of London, Regent’s Park, London, NW1 4RY UK; 9 Unit for Environmental Sciences and Management, North-West University, Potchefstroom, 2520 South Africa

**Keywords:** Antimicrobial peptide, Bufadienolide, Indirect effect, Infectious diseases, Innate immunity

## Abstract

**Background:**

*Batrachochytrium dendrobatidis* (*Bd*) is the causative agent of chytridiomycosis, one of the major causes of worldwide amphibian biodiversity loss. Many amphibians exhibit skin-based chemical defences, which may play an important role against invading pathogens, but whether the synthesis of these chemical compounds is enhanced or suppressed in the presence of pathogens is largely unknown. Here we investigated direct and indirect effects of larval exposure to the globally distributed and highly virulent *Bd-GPL* strain on skin secreted chemical defences and life history traits during early ontogeny of agile frogs (*Rana dalmatina*) and common toads (*Bufo bufo*).

**Results:**

Exposure to *Bd* during the larval stage did not result in enhanced synthesis of the antimicrobial peptide Brevinin-1 Da in *R. dalmatina* tadpoles or in increased production of bufadienolides in *B. bufo* tadpoles. However, exposure to *Bd* during the larval stage had a carry-over effect reaching beyond metamorphosis: both *R. dalmatina* and *B. bufo* froglets contained smaller quantities of defensive chemicals than their *Bd*-naïve conspecifics in the control treatment. Prevalence of *Bd* and infection intensities were very low in both larvae and metamorphs of *R. dalmatina*, while in *B. bufo* we observed high *Bd* prevalence and infection intensities, especially in metamorphs. At the same time, we did not find a significant effect of *Bd*-exposure on body mass or development rate in larvae or metamorphs in either species.

**Conclusions:**

The lack of detrimental effect of *Bd*-exposure on life history traits, even parallel with high infection intensities in the case of *B. bufo* individuals, is surprising and suggests high tolerance of local populations of these two species against *Bd*. However, the lowered quantity of defensive chemicals may compromise antimicrobial and antipredatory defences of froglets, which may ultimately contribute to population declines also in the absence of conspicuous mass-mortality events.

## Background

Amphibians are among the most threatened vertebrate groups, with their populations declining worldwide [1–3]. Although there may be no single main cause of declines [3], diseases caused by infection with viral, bacterial or fungal agents are clearly among the most devastating factors [4–6]. Studies aiming at uncovering and better understanding the processes that take place during interactions between amphibian hosts and their pathogens are therefore in the focus of current conservation-oriented research [7–9].

Chytridiomycosis, a disease affecting amphibians, is caused by the chytrid fungi *Batrachochytrium dendrobatidis* (*Bd*) and *Batrachochytrium salamandrivorans* (*Bsal*) [10]. Because *Bsal* has only been discovered recently [11], we know comparatively little about it [12], so that here we concentrate on chytridiomycosis caused by *Bd*. Chytridiomycosis has already led to the decline or extinction of several hundred species [13] and continues to cause mass mortality events on five continents due to repeated introductions arising from human activities [14]. *Bd* infects keratinous epidermal layers of the skin [15] and impairs its osmoregulatory function. This effect can cause shifts in electrolyte balance leading to cardiac asystolic death in juveniles and adults [16]. Tadpoles exhibit keratinous elements only in their mouthparts, so that they are less susceptible to *Bd* infection than later life-stages [17, 18], and can act as reservoirs in natural habitats [19–21].

As a part of innate immune defences, amphibians de novo synthesise numerous chemical compounds in their skin, serving as the first line of defence against pathogens and parasites [22]. These compounds can be cytolytic peptides, steroids, alkaloids or biogenic amines [23–27]. The most widespread defences against pathogens are cytolytic antimicrobial peptides (AMPs) which have been reported for species across eleven anuran families [27]. These AMPs are active against viruses, bacteria and microscopic fungi, including *Bd* [27–31]. The susceptibility of amphibian species and populations to chytridiomycosis is related to differences in AMP profiles [32, 33]. Bufonid toads lack skin-secreted AMPs [22], but may instead produce bufadienolides from early larval development on [34–36]. These steroid compounds exhibit antimicrobial, antiprotozoal activity, and may protect toads also against *Bd* [37, 38].

In this study, our aim was to experimentally investigate whether exposure to *Bd* resulted in increased production of skin-borne chemical defences as expressions of phenotypic plasticity, or if it caused lowered synthesis of defensive chemicals due to costs of infection or because of immune suppression. Therefore we exposed agile frog (*Rana dalmatina*; Fitzinger 1838) and common toad (*Bufo bufo*; Linnaeus, 1758) larvae to a highly virulent *Bd* isolate and monitored consequences for chemical defences and life history traits in well-developed tadpoles. Potential effects of larval infection reaching beyond metamorphosis on the abovementioned parameters were also tested. Adult *R. dalmatina* individuals produce at least one skin secreted AMP, Brevinin-1 Da [39], and *B. bufo* secrete several bufadienolide compounds in the skin [35, 36, 40]. Therefore, these species are suitable for the investigation of interactions between *Bd* infection and chemical defences.

## Results

### Rana dalmatina

**Fig. 1:**
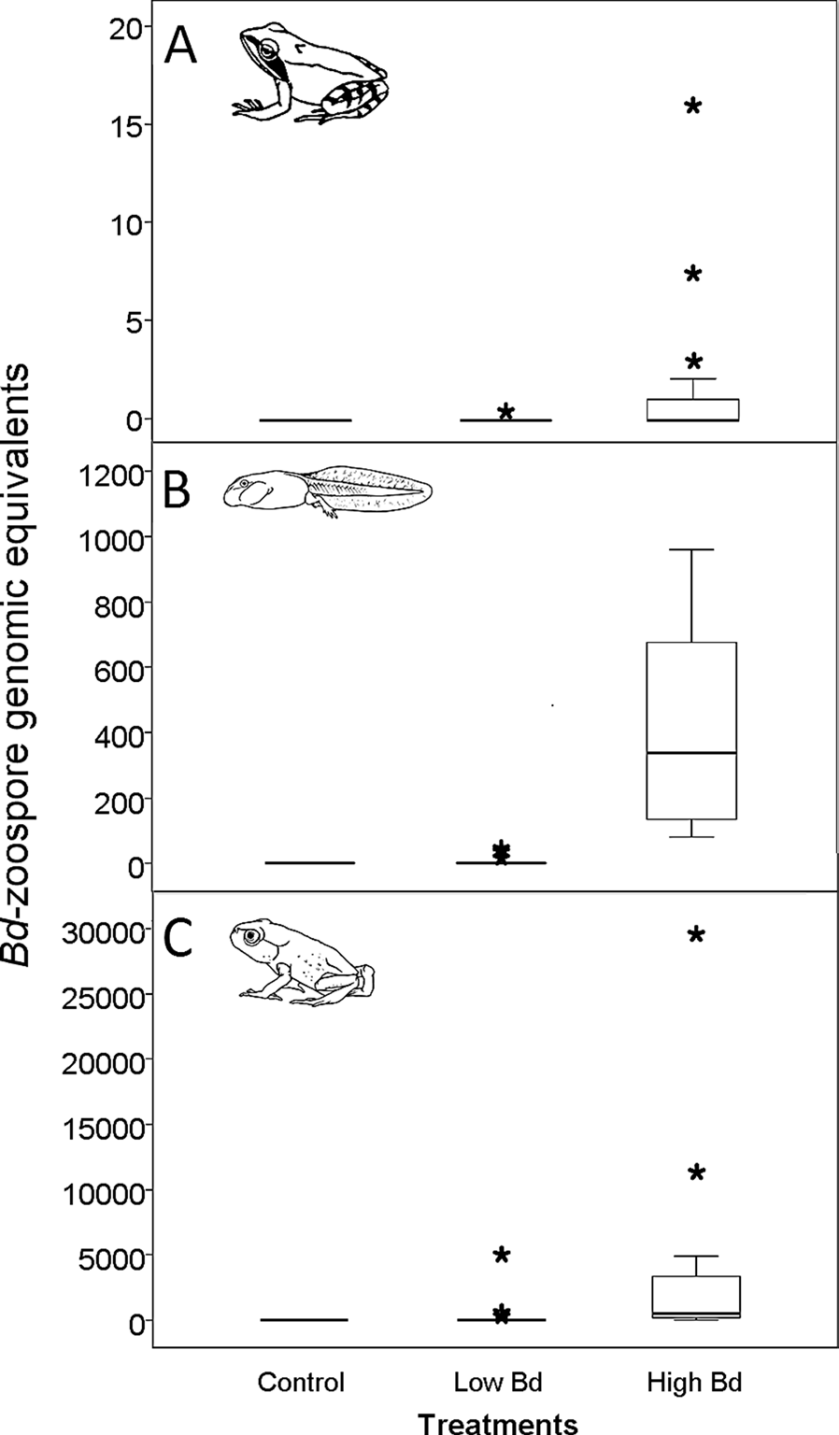
*Bd* Load as zoospore genomic equivalents in case of *Rana dalmatina* froglets (**A**) *Bufo bufo* tadpoles (**B**) and *B. bufo* toadlets (**C**). Tadpoles of *R. dalmatina* are not depicted because of extremely low *Bd* prevalence (one tadpole infected in the low *Bd* treatment and three tadpoles infected in the high *Bd* treatment). Note that scales are different. Horizontal lines represent medians, boxes represent interquartiles, bars represent ranges, asterisks indicate outliers (deviating from the boundary of the interquartile range (IQR) by more than 1.5 × IQR)

Control individuals all remained uninfected. The low *Bd* dose treatment resulted in very low infection prevalence and intensity in both tadpoles and froglets, while the high *Bd* dose treatment resulted in higher prevalence and intensity values in tadpoles and especially so in froglets (Table 1; Fig. 1A).

**Table 1:** Prevalence of *Bd* infection in the studied species after experimental exposure to *Bd*. N_tot_ is the total number of *Bd*-exposed individuals surviving until sampling, N_inf_ is the number of infected individuals at sampling. Control individuals are not shown because all remained uninfected

Species	Life stage	Treatment	N_tot_	N_inf_	Prevalence (%)
*Rana dalmatina*	Tadpoles	Low *Bd* dose	18	1	6
		High *Bd* dose	18	3	17
	Froglets	Low *Bd* dose	16	1	6
		High *Bd* dose	17	8	47
*Bufo bufo*	Tadpoles	Low *Bd* dose	18	6	33
		High *Bd* dose	17	17	100
	Toadlets	Low *Bd* dose	12	6	50
		High *Bd* dose	12	12	100

**Fig. 2:**
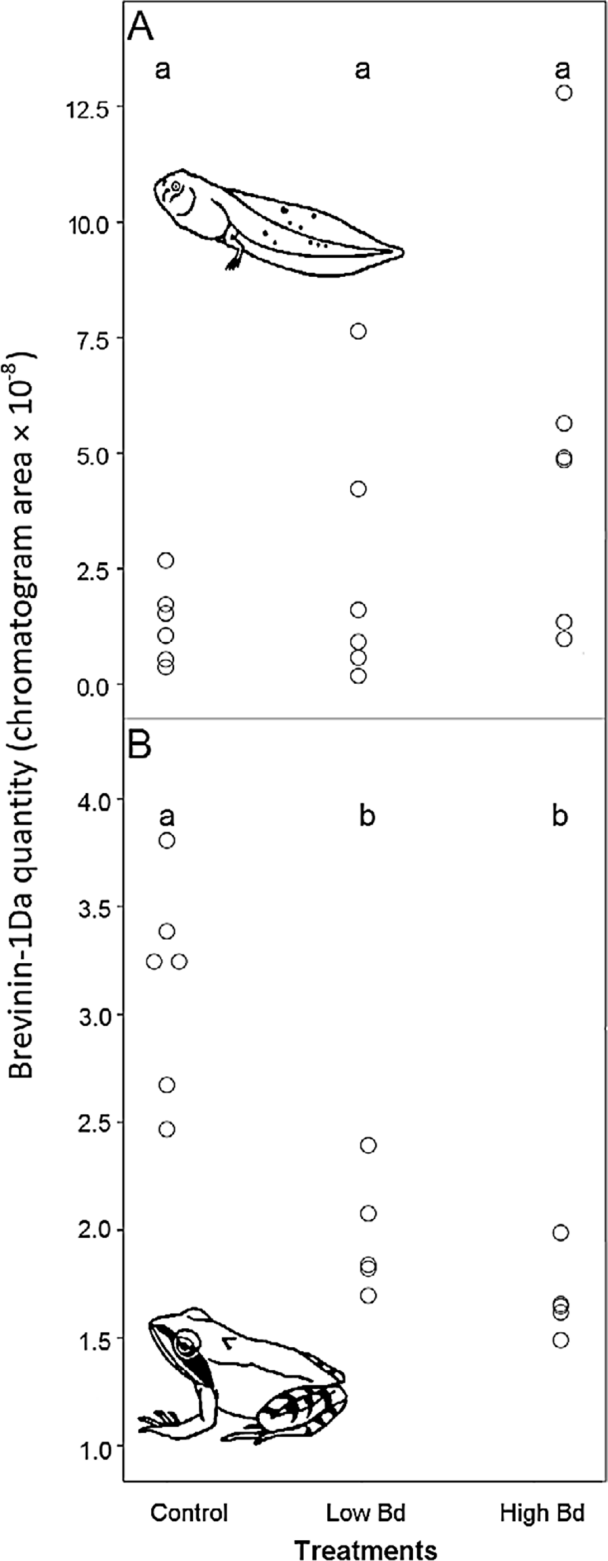
Quantity of the antimicrobial peptide Brevinin-1 Da in pooled samples of *R. dalmatina* tadpoles (**A**) and froglets (**B**). To obtain detectable quantities of AMP, we had to pool groups of three samples during sample preparation preceding chemical analysis. Letters in lower case indicate homogeneous subsets according to Tukey HSD post-hoc tests. Two overlapping data points are depicted next to each other in panel B in the control treatment. Note that in froglets the low *Bd* dose and the high *Bd* dose treatment contained only 5 replicates as opposed to 6 replicates in the other groups

Treatment had no effect on the relative amount of Brevinin-1 Da in pooled samples of tadpoles (GLM: *F*_2,15_ = 2.31, *P* = 0.13; Fig. 2A). However, older tadpoles tended to exhibit larger amounts of the peptide than younger conspecifics (*F*_1,16_ = 4.51, *P* = 0.05). Infection intensity and body mass were not related to Brevinin-1 Da quantity (infection intensity: *F*_1,16_ = 1.14, *P* = 0.30; body mass: *F*_1,16_ = 0.67, *P* = 0.43). In case of froglets, however, exposure to *Bd* resulted in significantly reduced relative amounts of Brevinin-1 Da in both *Bd* treatments as compared to the controls (GLM: *F*_2,13_ = 30.60, *P* < 0.001; Fig. 2B). The other measured variables had no detectable effect on Brevinin-1 Da quantity (length of larval development: *F*_1,12_ = 0.37, *P* = 0.55; infection intensity: *F*_1,12_ = 0.35, *P* = 0.57; body mass: *F*_1,12_ = 3.31, *P* = 0.09).

Body mass of tadpoles was positively affected by development stage, but treatment had no significant effect on it, either alone, or in interaction with development stage. In case of froglets, treatment had a marginally non-significant effect on body mass, where individuals exposed to the high *Bd* dose treatment tended to be heavier than others. Length of larval development had no effect on body mass, either alone or in interaction with treatment (For details see Table 2). Treatment had no significant effect on development, either measured as tadpoles’ development stage at sampling or as the length of larval development in case of froglets (Table 2).

**Table 2:** The effect of treatments on development and body mass of individuals at two life stages in the two studied species. Results are based on General Linear Models. Significant differences are highlighted in bold

Species	Life stage	Dependent variables	Explanatory variables	*B*	*SE*	*df*	*F*	*P*
*Rana dalmatina*	Tadpoles	Development stage	Treatment			2, 50	0.36	0.70
		Body mass	**Development stage**	**0.06**	**0.01**	**1, 51**	**44.51**	** < 0.001**
			Treatment			2, 49	0.84	0.44
			Treatment × development stage			2, 47	1.49	0.24
	Froglets	Length of larval development	Treatment					0.95*
		Body mass	Length of larval development	− 1.63	1.61	1, 49	1.02	0.31
			Treatment			2, 48	3.13	0.05
			Treatment × length of larval development			2, 45	0.89	0.42
*Bufo bufo*	Tadpoles	Development stage	Treatment			2, 50	1.07	0.35
		Body mass	**Development stage**	**26.02**	**4.06**	**1, 51**	**41.11**	** < 0.001**
			Treatment			2, 49	0.25	0.78
			Treatment × development stage			2, 47	0.88	0.42
	Toadlets	Length of larval development	Treatment			2, 35	0.03	0.97
		Body mass	**Length of larval development**	**− 0.19**	**0.01**	**1, 36**	**9.87**	**0.003**
			Treatment			2, 34	0.95	0.40
			Treatment × length of larval development			2, 32	0.92	0.41

*Result based on Kruskal–Wallis test

### Bufo bufo

None of the control individuals were infected, but we obtained relatively high infection prevalence and intensities in toadlets exposed to the low *Bd* dose treatment and in both life stages exposed to the high *Bd* dose treatment (Table 1; Fig. 1B and C).

**Fig. 3:**
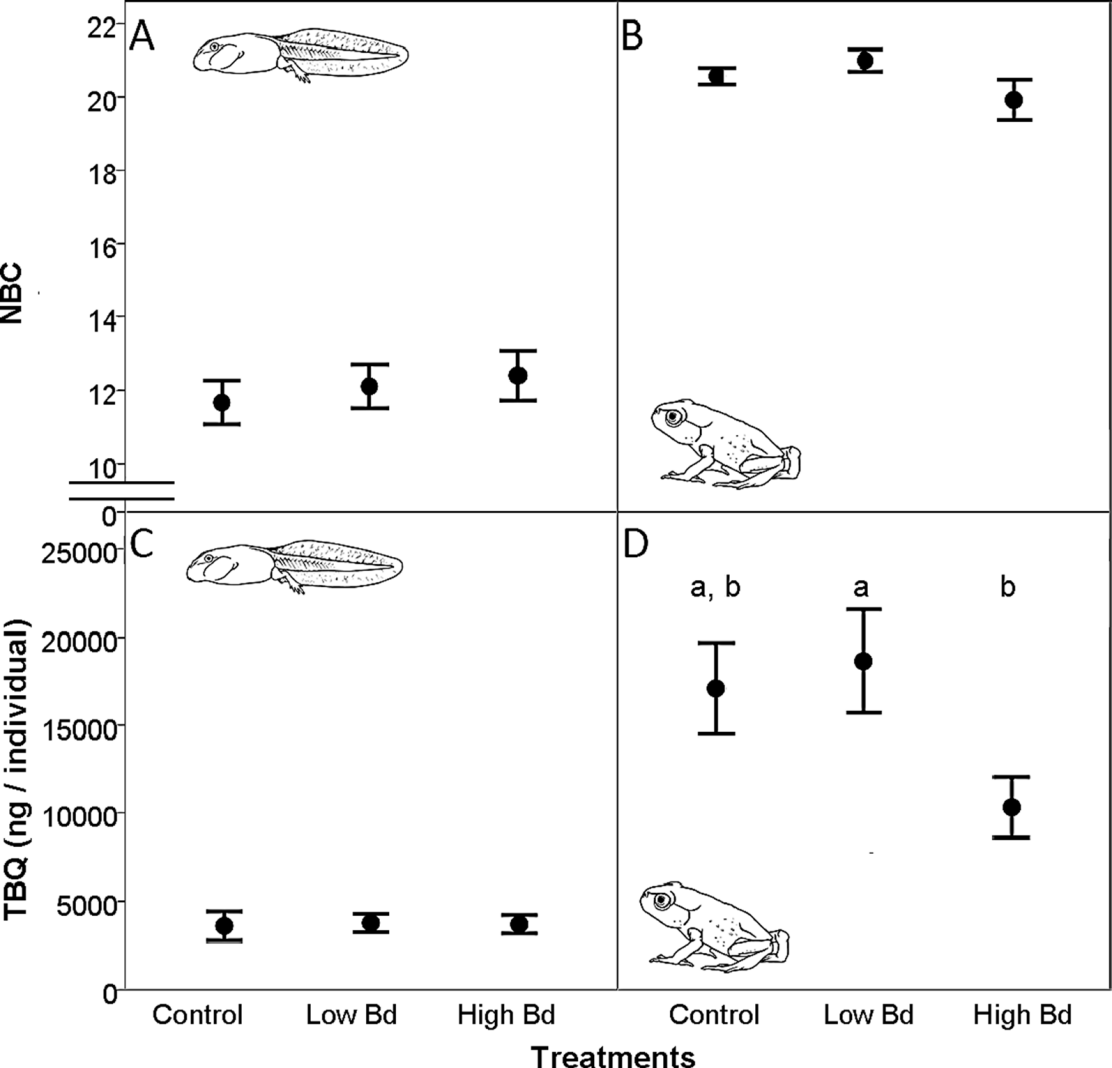
Toxin content of *B. bufo* larvae and toadlets in the control and *Bd*-exposure treatments: Number of bufadienolide compounds (NBC; mean ± SE) in tadpoles (**A**) and toadlets (**B**), and total bufadienolide quantity (TBQ; mean ± SE) in tadpoles (**C**) and toadlets (**D**) after exposure to zero (control), low or high *Bd* zoospore concentrations. Note that the scale is not continuous in case of NBC. Letters in lower case indicate homogeneous subsets according to Tukey HSD post-hoc tests

We detected 22 different bufadienolide compounds in *B. bufo* extracts, three of which we identified with the help of the standards as arenobufagin, telocinobufagin and bufotalin (Table 3). The presence of individual compounds showed varied age-dependent patterns: some bufadienolides were present at both life stages in all (e.g., compound 7) or nearly all individuals (e.g., compound 13), while others occurred in a high proportion of individuals only after metamorphosis (e.g., compounds 10–12; Table 3). After metamorphosis, toadlets produced more bufadienolide compounds and also experienced a two to three fold increase in TBQ compared to tadpoles (Fig. 3).

**Table 3:** Percentages of *Bufo bufo* individuals that contained various bufadienolide compounds in the two development stages. We could unambiguously identify three compounds based on the standards and detected another 19 compounds as unknown bufadienolides based on their characteristic UV spectra. Analytical properties of the detected compounds are provided. (N: sample size (number of individuals), m/z: mass/charge)

Bufadienolide compounds	Percentage of individuals containing the compound		Analytical properties	
	Tadpoles (N = 53) (%)	Toadlets (N = 38) (%)	Retention time (minute)	m/z (M+H^+^)
Arenobufagin	59	74	4.8	417
Telocinobufagin	34	100	8.8	403
Bufotalin	66	100	9.8	445
Compound 1	9	53	4.1	615
Compound 2	17	100	4.7	699
Compound 3	81	100	5.0	417
Compound 4	26	95	5.8	713
Compound 5	0	58	6.0	601
Compound 6	51	100	6.1	415
Compound 7	100	100	7.0	729
Compound 8	49	100	7.5	701
Compound 9	100	100	8.0	727
Compound 10	0	100	9.3	715
Compound 11	0	100	10.6	713
Compound 12	19	100	11.1	401
Compound 13	96	95	11.3	715
Compound 14	0	100	14.9	701
compound 15	100	95	17.5	757
Compound 16	98	84	19.3	573
Compound 17	100	100	20.5	571
Compound 18	100	97	22.1	367
Compound 19	100	100	23.3	365

The number of bufadienolide compounds (NBC) did not differ among treatments in either life stage (GLM; tadpoles: *F*_2,49_ = 0.49, *P* = 0.62; toadlets: N = 38, *F*_2,35_ = 1.6, *P* = 0.22; Fig. 3 A and B). NBC was positively related to dry mass in tadpoles (*B* = 0.16, *SE* = 0.08, *F*_1,51_ = 4.56, *P* = 0.038), but not in toadlets (*F*_1,36_ = 1.78, *P* = 0.19). Infection intensity and development had no significant effect on NBC either in tadpoles (infection intensity: *F*_1,50_ = 1.82, *P* = 0.18; development stage: *F*_1,50_ = 0.002, *P* = 0.97) or in toadlets (infection intensity: *F*_1,36_ = 2.31, *P* = 0.13; length of larval development: *F*_1,36_ = 0.02, *P* = 0.88).

Total bufadienolide quantity (TBQ) was not affected by treatment in *B. bufo* tadpoles (GLM; *F*_2,50_ = 0.31, *P* = 0.74; Fig. 3C), but differed significantly among treatments in case of toadlets (*F*_2,34_ = 4.08, *P* = 0.026; Fig. 3D). TBQ was not related to dry mass (*F*_1,51_ = 10.68, *P* = 0.20), but this relationship was positive in case of toadlets (*B* = 2.21, *SE* = 0.69, *F*_1,34_ = 10.39, *P* = 0.003). According to Tukey HSD post hoc tests on the residuals of the regression of TBQ on toadlet dry mass (*R* = 0.47, *F*_1,37_ = 10.06, *P* = 0.003), relative TBQ was lower by 23% in the high *Bd* dose treatment than in the low *Bd* dose treatment (Mean difference = − 33.31, *SE* = 11.93, *P* = 0.022), but these two treatment groups did not differ from the control (high *Bd* dose: Mean difference = − 23.99, *SE* = 11.49, *P* = 0.11; (low *Bd* dose: Mean difference = 9.32, *SE* = 11.49, *P* = 0.7; Fig. 3D). Infection intensity and development stage did not have an effect on NBC either in tadpoles (infection intensity: *F*_1,51_ = 0.69, *P* = 0.41; development stage: *F*_1,51_ = 0.04, *P* = 0.85) or in toadlets (infection intensity: *F*_1,33_ = 0.038, *P* = 0.85; length of larval development: *F*_1,33_ = 1.46, *P* = 0.24).

Body mass was affected by development stage in case of tadpoles: individuals that were less developed also had a lower body mass. Treatment and its interaction with development stage had no significant effect on body mass (Table 2). Within the high *Bd* dose treatment, where *Bd* prevalence was sufficiently high to be analyzed, tadpole body mass was again positively related to development stage (*F*_1,15_ = 29.6, *P* < 0.001), while infection intensity had no effect on mass (GLM: *F*_1,14_ = 0.003, *P* = 0.96). Fourteen days after completion of metamorphosis, body mass of toadlets was in a negative relationship with the length of larval development (Table 2). Treatment and its interaction with the length of larval development had no significant effect on toadlet body mass (Table 2). Within the low *Bd* dose treatment group, neither infection intensity, nor the length of larval development had an effect on body mass of toadlets (GLM: infection intensity: *F*_1,10_ = 2.01, *P* = 0.19; length of larval development: *F*_1,10_ = 0.38, *P* = 0.55). However, in the high *Bd* dose treatment, infection intensity had a significant negative effect (GLM: *B* = − 4.38, *SE* = 1.73, *F*_1,10_ = 6.39, *P* = 0.03), and the length of larval development a marginally significant positive effect (*B* = − 5.01, *SE* = 2.28, *F*_1,9_ = 5.01, *P* = 0.052) on toadlet body mass. Development was not affected by treatment at either life stage (Table 2), and it was not related to infection intensity in either one of the assessed treatment groups (Spearman correlation in case of tadpoles in the high *Bd* dose treatment: *R* = 0.025, N = 17, *P* = 0.93; Pearson correlations in case of toadlets: low *Bd* dose:* R* = − 0.301, N = 12, *P* = 0.34; high *Bd* dose:* R* = 0.157, N = 12, *P* = 0.63).

## Discussion

In the present study experimental exposure to *Bd* did not result in significantly increased production of the antimicrobial peptide Brevinin-1 Da in *R. dalmatina* tadpoles, nor did it influence bufadienolide toxin synthesis in *B. bufo* tadpoles. However, *Bd*-exposure during the larval stage negatively affected chemical defences in metamorphosed individuals of both species. These results suggest that neither larvae nor freshly metamorphosed individuals respond to *Bd*-exposure with enhanced synthesis of antimicrobial chemicals, and that infection during the larval stage may rather carries costs that manifest in decreased quantities of chemical defences in metamorphs. Our results further indicate that larvae of the agile frog (*R. dalmatina*) were resistant to infection with a highly virulent *Bd* isolate, as indicated by low prevalence and infection intensities. At the same time, tadpoles of the common toad (*B. bufo*) were not resistant, but tolerant, as suggested by high *Bd* prevalence and high infection intensities, but no malign effects on life history traits: exposure to *Bd* did not influence body mass or development rate in larvae or metamorphs in either species.

The knowledge available regarding the occurrence of AMP synthesis in larval anurans is limited and controversial. Skin-associated granular glands and their ducts are mostly immature before metamorphosis in most species [41, 42], suggesting no, or limited AMP production (but see [43]). However, at the same time, gland products may also be secreted by a merocrine process, where the secretum reaches the skin surface via exocytosis directly or through the epidermal interstitium [41, 44, 45]. Furthermore, the adaptive immune system is suppressed during metamorphosis to prevent immune responses against newly emerging tissue types [46], suggesting a greater reliance on innate immune defences against invading pathogens during this susceptible period. Schadich et al. [47] found no evidence of AMP synthesis in tadpoles of *Litoria ewingii* despite intense AMP production after metamorphosis, while Wabnitz et al. [48] demonstrated efficient AMP synthesis in the skin of *Litoria splendida* at both larval and adult life stages. While Woodhams et al. [49] did not find AMPs in an early larval stage (development stage 25 according to Gosner, [50]) in two closely related species, *Rana arvalis* and *R. temporaria*, we detected that *R. dalmatina* tadpoles produce de novo the same AMP as do adults [39] at least during late larval development (development stage 37 according to Gosner). Whether this discrepancy among studies indicates species-specific differences in AMP synthesis, or if AMP production starts in Ranids some time later during larval development remains to be determined.

Our expectation that AMP synthesis is boosted upon exposure to *Bd* in *R. dalmatina* was not met. Phenotypic plasticity in chemical defences is not well understood [51], but some studies suggest that environmental stressors such as competition [52] and pathogen presence [53, 54] can induce an increase in AMP synthesis in metamorphosed anuran amphibians. Furthermore, adult European water frogs (*Pelophylax lessonae* and *P. esculentus*) are capable of elevating AMP synthesis in response to *Bd*, if the skin microbiota is suppressed [55]. Although during the tadpole stage we also observed a slight increase in the relative amount of Brevinin-1 Da upon *Bd* exposure, this was not significant, and we detected a sharp reduction in AMP quantity in both *Bd*-exposed groups in froglets. Wild caught, infected adults of the frog *Litoria serrata* exhibited similarly reduced quantities of AMPs compared to uninfected conspecifics [56], but whether this was a cause or consequence of infection remained unknown. Our results suggest that reduced AMP expression can be a consequence of exposure to *Bd*. A reduced synthesis of AMPs may result from immunosuppression by *Bd*, as demonstrated in case of the adaptive immune system [57–59]. Lowered AMP production may also be a direct cost of infection, or may be indirectly caused by elevated corticosterone levels resulting from *Bd*-infection [60, 61] because elevated corticosterone levels can cause reduced AMP synthesis [62, 63]. Whether the reduced quantity of Brevinin-1 Da is still large enough to be effective against *Bd* in metamorphs, or other agents of the immune system can take over the role of Brevinin-1 Da in preventing severe infection requires further investigation.

Interestingly, AMP synthesis was reduced in metamorphs arising from the low *Bd* dose treatment without detectable amounts of *Bd* on all but one froglet. There are, however, precedents for significant effects of *Bd* exposure on various traits in the absence of confirmed infection. For example, Garner et al. [64] experienced significant mortality in *B. bufo* tadpoles and freshly metamorphosed individuals due to *Bd* exposure without detectable infection loads. Also, *Bd* exposure reduced growth in European treefrogs (*Hyla arborea*) without detectable levels of infection [65]. Individuals are probably able to prevent initial infections or naturally clear *Bd* infections acquired but may suffer the costs of the mounted immune response [66]. Alternatively, *Bd* presence in the surrounding aquatic environment may be sufficient to induce pathology or responses (e.g., via waterborne chemicals) even if infection does not occur, as shown in case of crayfishes (*Procambarus* spp. and *Orconectes virilis*) which are alternative hosts of *Bd* [67].

Exposure to *Bd* had no detectable effect either on the number of bufadienolide compounds or on total bufadienolide quantity in case of *B. bufo* tadpoles. Similarly, NBC of toadlets was also not affected by *Bd* presence. However, TBQ in the high *Bd* dose treatment was significantly lower compared to the low *Bd* dose treatment in toadlets. These results clearly indicate that *Bd*-prevalence and higher infection loads did not induce enhanced toxin synthesis. Besides their role in the chemical defence system, bufadienolides also contribute to the osmotic homeostasis of toads [68, 69]. The decreased TBQ in the high *Bd* dose may have resulted from a compensatory response to the altered electrolyte balance (reduced sodium and potassium concentrations) due to *Bd* infection [16], but this speculation needs experimental confirmation. Alternatively, *Bd* infection can lead to structural damage in the skin and its glands [10], which may have contributed to the lowered toxin production in the high *Bd* dose treatment. Whatever the cause is, heavily infected toadlets unable to produce the increase in toxin content after metamorphosis, may suffer from detrimental consequences because skin secreted bufadienolides can act as repellents against vertebrate predators [70, 71], they play a role in immune defence [37, 38, 72] and are important for osmotic homeostasis [68, 69]. Lowered TBQ in the high *Bd* dose treatment, thus, indicates a possible indirect negative effect of *Bd* infection, similarly to what we found in regard to the chemical defence of *R. dalmatina*.

Exposure to *Bd* did not affect the measured life history traits in either species at the tadpole stage. In larval anurans only the mouthparts are keratinized structures [17], thus, mortalities due to chytridiomycosis are rare and susceptibility varies among species [10, 18, 73, 74]. However, sublethal negative effects of *Bd* infection can also occur due to mouthpart damage [75, 76], lethargy and poor swimming performance, resulting in lowered body mass and growth [18, 74, 76, 77]. In the present study only a very few tadpoles of *R. dalmatina* became infected and in these individuals infection intensity was very low in both *Bd* treatments. In case of *B. bufo* tadpoles, prevalence of infection and infection intensities were high, especially in the high *Bd* dose treatment, so that the lack of fitness consequences was somewhat surprising. Two out of the three identified bufadienolide compounds (arenobufagin and telocinobufagin) were previously documented to moderately inhibit the growth of *Bd* [38]. These and some of the other unidentified bufadienolide compounds may have contributed to the high *Bd*-tolerance of *B. bufo* individuals, which lack AMPs. These results suggest that the studied populations exhibit low susceptibility to *Bd* infection during larval development: tadpoles of *R. dalmatina* appear to be highly resistant, while *B. bufo* larvae may be highly tolerant.

During metamorphic climax, *Bd* starts to colonise newly keratinized skin surfaces and spreads out on the entire animal [17, 78]. In the present study, both the prevalence of *Bd* (GPL, IA042) and infection intensities were high in case of *B. bufo*, which is consistent with previous studies that used the same isolate and resulted in significant negative effects on body mass and mortality of *B. bufo* originated from the United Kingdom [64, 79]. Mortalities due to chytridiomycosis in the closely related *B. spinosus* (formerly a subspecies of *B. bufo*) were also observed in Spain [64, 80]. Contrary to this, the length of larval development was not affected by *Bd* exposure in our experiment, and we did not observe significant negative effects on body mass of toadlets. Only in the high *Bd* dose treatment, infection intensity was negatively related to body mass: lighter individuals had higher infection intensities, than heavier ones. Whether this pattern was a cause or consequence of infection remains unclear. All in all, these results suggest that *B. bufo* individuals of the studied population in Central Europe may be more tolerant to *Bd* than those in Western European populations. Alternatively to this spatial hypothesis, the pattern may also arise due to temporal differences; toads may have adapted to the presence of *Bd* since the earlier studies [64, 79, 80]. Furthermore, populations may respond differently to *Bd* infection from year to year due to phenotypic plasticity or epigenetic changes. These processes could also have contributed to the observed differences between the present and former studies in toad susceptibility. Regarding *R. dalmatina*, we detected low prevalence of infection in the low *Bd* dose treatment and moderate prevalence with low infection intensities in the high *Bd* dose treatment, and no adverse effects of *Bd-*exposure on life history traits of froglets. These results are in line with those of previous field studies suggesting that *R. dalmatina* is resistant to chytridiomycosis [21, 81, 82]. The less keratinized skin as well as the presence of Brevinin-1 Da may make the skin of *R. dalmatina* less suitable for *Bd* growth, hence the lower probability and intensity of infection as compared to *B. bufo*, which speculations need further investigations in the future.

## Conclusions

Our results provide evidence that exposure to *Bd* can have negative effects on two different types of chemical defences of phylogenetically distant species in a later life-stage, even in the absence of obvious immediate effects on life-history traits, or, indeed, actual *Bd* infection. Because the investigated chemical defences are widespread among amphibians, the results of this study are likely to be applicable to many species with similar chemical defences, and this hidden effect of *Bd* presence in aquatic habitats should be considered in the future. Whether weakened chemical defences lead to lowered fitness in affected individuals remains an open question that will need further investigation.

## Methods

### Experimental procedures

In March 2016, we collected 40 eggs from each of nine freshly laid egg clutches of *R. dalmatina* from a pond in the Pilis-Visegrádi-Hills, Hungary (47.767058 N, 18.981325 E). We transported them to the Experimental Station Júliannamajor of the Plant Protection Institute, Centre for Agricultural Research. The Közép-Duna-Völgyi KTVF issued the permission to conduct the study (PE/KTF:3596-6-8/2016) and the Ethical Commission of the ATK NÖVI approved the investigation in accordance with Good Scientific Practice guidelines and national legislation. We placed eggs from each clutch separately into plastic boxes (24 × 16 × 13 cm) holding 1 L of reconstituted soft water (RSW; [83]) at a constant temperature of 19 °C and a 12:12 h light:dark cycle. Eggs were disinfected by bathing them for 3 days in 10 mg/L chloramphenicol in order to prevent accidental *Bd* infection [84] because *Bd* is reportedly present in the study area [21], even if with low prevalence [82]. Although chloramphenicol is an antibiotic agent, it is also effective against *Bd* [84, 85] and can be safely used for the disinfection of amphibian eggs [86].

Five days after hatching, when larvae were at development stage 25 (Gosner), we started the experiment with 12 healthy-looking tadpoles from each family. We maintained tadpoles individually in plastic boxes (15 × 12 × 12 cm) filled with 1 L of RSW, and fed tadpoles with slightly boiled and smashed spinach complemented with *Spirulina* powder (1 m/m%) ad libitum. We changed water twice a week using different dip nets for each treatment to prevent contamination across treatments. Temperature was 19.4 ± 0.7 °C (mean ± SD) during the experiment. The light:dark cycle was adjusted weekly to outdoor conditions, starting with 12:12 h light:dark in late March which we gradually changed to 14:10 h by the end of April. We exposed tadpoles during the entire larval development to sterile culture broth (control), or to a low or high zoospore concentration in liquid *Bd* culture (low *Bd* dose and high *Bd* dose treatments hereafter; for details, see below). We assigned tadpoles to the treatments using stratified randomization, and arranged rearing boxes into randomized spatial blocks, each containing one replicate from each treatment. We exposed individuals of nine families to the three treatments in four replicates, which resulted in a total of 108 experimental units. Hatchlings that were not used in the experiment were released at the site of origin.

Thirty days after start of the experiment, we randomly selected half of the tadpoles, sampled their skin secretions (for details see below) then gently blotted them dry and weighed them to the nearest mg using an OHAUS-PA213 analytical balance. Thereafter, we euthanized and preserved tadpoles in 70% ethanol and stored samples at 4 °C until further analysis.

We monitored development of remaining tadpoles daily. When an individual reached development stage 42 (emergence of forelimbs; according to Gosner) we poured the water off, placed back the tadpole into the same box covered with a transparent and perforated lid, added 100 ml RSW and lifted one side of the container by ca. 2 cm to provide metamorphs with both a body of water and a dry surface. Once metamorphs reached stage 46 (complete tail resorption; according to Gosner) we placed individuals into new, covered boxes of the same size as before, equipped with wet paper towels and a piece of cardboard egg-holder as a shelter. We fed the froglets with small crickets (*Acheta domestica*, instar stage 1–2) ad libitum. Dates of metamorphosis and of completion of tail resorption were registered daily. Fourteen days after completion of tail resorption we weighed animals and humanely euthanized them using the “cooling then freezing” method [87], preserved individuals in 70% ethanol and kept samples at 4 °C until further processing.

We conducted the same experiment using individuals of *B. bufo*. However, we observed high mortality independently from treatments (15 out of 36 tadpoles died both in the control and in the low *Bd*-dose treatment, and 10 out of 36 tadpoles died in the high *Bd*-dose treatment), presumably due to a bacterial bloom caused by the simultaneous presence of *Spirulina* and culture broth (an effect we did not observe in case of *R. dalmatina* tadpoles in this experiment, or when tadpoles were fed solely with spinach in previous experiments). Consequently, 17 days after start of the first experiment, we re-started this part of the study and conducted the same experiment as described above with 108 randomly chosen *B. bufo* tadpoles from the same location with the following additional differences: (1) Eggs originating from 14 different clutches were kept in mixed groups in outdoor mesocosms containing 130 L of aged tap water, 40 g beech leaves and 0.5 L pond water with no prior chloramphenicol treatment. (2) Once brought into the laboratory after hatching, we fed tadpoles with smashed and slightly boiled spinach only (no *Spirulina*). (3) Light exposure adjustment was different because of the delayed start (4) Because of their smaller size, we fed toadlets with springtails (*Folsomia* sp.) after metamorphosis.

### Maintenance of *Bd* culture and experimental exposure

We experimentally infected tadpoles with the global pandemic lineage (GPL) of *Bd*. This isolate originated from a dead *Alytes obstetricans* (IA042) collected in 2004 from a mass mortality event in Spanish Pyrenees. Cultures were maintained in mTGhL broth (8 g tryptone, 2 g gelatine-hydrolysate and 4 g lactose in 1000 ml distilled water) in 25 cm^2^ cell culture flasks at 4 °C and passed every three months into sterile mTGhL. One week before use, we inoculated 100 ml mTGhL broth with 1–2 ml of these cultures in 175 cm^2^ cell culture flasks and incubated them for seven days at 22 °C. We assessed the concentration of intact zoospores using a Bürker chamber at × 400 magnification. During inoculation of tadpoles’ rearing boxes, the mean initial concentrations were ~ 1.8 × 10^6^ (used to infect *R. dalmatina*) and ~ 2 × 10^6^ (used to infect *B. bufo*) zoospores (zsp)/ml in the flasks. These cultures were used for the high *Bd* dose treatment and we prepared a 100-fold dilution with sterile mTGhL broth for the low *Bd* dose treatment. After each water change, we inoculated 1 ml of these cultures into the tadpoles’ rearing boxes holding 1 L RSW, resulting in 18–20 zsp/ml in the low *Bd* dose treatment and 1800–2000 zsp/ml in the high *Bd* dose treatment. Similar zoospore concentrations have been used widely and successfully in studies involving experimental infection [18, 75, 88]. We inoculated controls with the same quantity of sterile mTGhL broth. Contaminated water and equipment were disinfected overnight with VirkonS before disposal [89].

### Assessment of infection intensity using qPCR

We assessed infection intensity from dissected mouthparts in case of preserved tadpoles and from toe clips in case of metamorphs. We homogenized tissue samples, extracted DNA using PrepMan Ultra Sample Preparation Reagent (Thermo Fisher Scientific, Waltham, Massachusetts, USA) according to previous recommendations [90], and stored extracted DNA at − 20 °C until further analyses. We assessed infection intensity using real-time quantitative real-time polymerase chain reaction (qPCR) following a standard amplification methodology targeting the ITS-1/5.8S rDNA region [90] on a BioRad CFX96 Touch Real-Time PCR System. To avoid PCR inhibition by ingredients of PrepMan, samples were diluted ten-fold with double-distilled water. We ran samples in duplicate. In case the result was equivocal, we repeated reactions in duplicate. If it again returned an equivocal result, we considered that sample to be *Bd* positive [91]. Genomic equivalent (GE) values were estimated from standard curves based on four dilutions of a standard (100, 10, 1 and 0.1 zoospore genomic equivalents; provided by J. Bosch; Museo Nacional de Ciencias Naturales, Madrid, Spain).

### Skin secretion sampling in *R. dalmatina* and analysis of Brevinin-1 Da

We analysed skin secretions in two ontogenetic stages: 30 days after start of the experiment, when larvae were in development stage 36.9 ± 1.4 (mean ± SD; according to Gosner) and 14 days after completion of metamorphosis. Following the procedure described in [92] we collected skin secretions non-invasively by bathing tadpoles or froglets individually in 5 ml polypropylene tubes containing 5 ml collection buffer with 0.1 mM norepinephrine (NE) bitartrate for 15 min. After removing animals, we acidified the NE solution by adding 50 µL of 99% trifluoracetic acid (TFA), to reach a final concentration of 1 V/V%. Samples were stored at − 20 °C until further analyses. As indicated by preliminary assessments of Brevinin-1 Da concentrations, we had to pool groups of three samples within treatments to obtain detectable quantities of the targeted AMP. As a pre-step of purification, we activated each reverse-phase Sep-Pak cartridges (200 mg, LiChrolut RP-18, Merck-Millipore) with 2 ml acetonitrile and subsequently rinsed with 2 ml solvent A (HPLC-grade water with 0.12 V/V% TFA; according to [93]). Next, we loaded the skin extracts onto the cartridges, saved the solution, rinsed cartridges with 2 ml solvent A, and loaded the saved solution onto the cartridges again. Finally, we rinsed cartridges with 4 ml solvent A. We eluted the purified skin secretion with 2 ml solvent B (70:30 acetonitrile:HPLC grade water, acidified with TFA to a final concentration of 0.1 V/V%) into 2 ml polypropylene tubes and dried samples using a vacuum centrifuge (Savant, Integrated Speed Vac System, ISS 100).

We accomplished peptide identification and quantification using nano-UHPLC-MS/MS liquid chromatography-mass spectrometry, with a Maxis II ETD QqTOF (Bruker Daltonics, Bremen, Germany) coupled to an Ultimate 3000 nanoRSLC system (Dionex, Sunnyvale, CA, USA) under the control of Hystar v.3.2 (Bruker Daltonics, Bremen, Germany). We dissolved samples in 2% acetonitrile and 0.1% formic acid in water, out of which 5 µl were injected onto an Acclaim PepMap100 C-18 trap column (100 µm × 20 mm, Thermo Scientific, Sunnyvale, CA, USA). We performed sample desalting and preconcentration with 0.1% TFA for 8 min with a flow rate of 5 µl/min. Peptides were separated on an ACQUITY UPLC M-Class Peptide BEH C18 column (130 Å, 1.7 µm, 75 µm × 250 mm, Waters, Milford, MA, USA) at 48 °C, using a flow rate of 300 nl/min. We used the following HPLC solvents: solvent A containing 0.1% formic acid in water and solvent B containing 0.1% formic acid in acetonitrile, with the gradient: 4% B from 0 to 11 min, followed by a 120 min gradient to 50% B, then elevated the concentration of solvent B to 90% in 1 min and kept it there for 10 min. After each sample a blank was run to avoid carry-over. Sample ionization was achieved in the positive electrospray ionization mode via a CaptiveSpray nanoBooster ion source with capillary voltage set to 1300 V, at 0.2 Bar nanoBooster pressure. The drying gas was heated to 150 °C with 3 L/min flow rate. For external mass calibration, we used the low concentration tuning mix from Agilent technologies via direct infusion. Internal mass calibration was performed via lock mass for each run using sodium formate with the following ion transfer parameters: prepulse storage 10 µs, collision transfer 10 µs, quadrupole ion energy 5 eV, Funnel 1 RF 400 Vpp, Multipole RF 400 Vpp. The collision RF was set to 1200 Vpp with 120 µs ion transfer time. We identified the chromatographic peaks as Brevinin-1 Da by comparing retention time and mass spectrum of the 453.3551^4+^ fragment to a commercially purchased molecular standard (Biocenter Kft., Szeged, Hungary). We analysed chromatograms using the software Compass Data Analysis (version 4.0, Bruker daltonics Inc., Billerica, USA) to obtain chromatogram area values as quantity estimates for statistical analysis.

### Skin toxin sampling in *B. bufo* and analysis of bufadienolides

We collected bufadienolide samples from tadpoles preserved at development stage 36.3 ± 0.9 (mean ± SD; according to Gosner) and from 14 day old toadlets (that finished metamorphosis 14 days earlier). We homogenized whole bodies with a homogenizer (VWR VDI 12) equipped with a dispersing tool (IKA S12N-7S), dried samples under vacuum at 45 °C using a rotary evaporator (Büchi Rotavapor R-134, Flawil, Switzerland), weighed dry mass (dry body mass henceforth) and re-dissolved samples in 1 ml absolute HPLC-grade methanol, aided by brief exposure to ultrasound in a bath sonicator (Tesla UC005AJ1). As the last step of sample preparation, we filtered samples through FilterBio nylon syringe filters (pore size = 0.22 μm) and stored them at − 20 °C until further analyses.

We analysed bufadienolide compounds by means of high-performance liquid chromatography coupled with diode-array detector and electrospray ionization mass spectrometry (HPLC–DAD-ESI–MS). We identified the chromatographic peaks as bufadienolides based on the UV spectrum [34] and by comparing their retention time and mass spectrum to those of the following commercially available standards: bufalin, bufotalin, resibufogenin, gamabufotalin, areno- and telocinobufagin (Biopurify Phytochemicals, Chengdu, China), cinobufagin (Chembest, Shanghai, China), cinobufotalin (Quality Phytochemicals, New Jersey, USA) and digitoxigenin (Santa Cruz Biotechnology, Dallas, TX, USA) or to compounds established by analysing a large sample obtained from an adult male common toad by gently massaging the parotoid glands.

We performed HPLC–MS measurements on a Shimadzu LC–MS 2020 instrument (Shimadzu, Kyoto, Japan) that consisted of a binary gradient solvent pump, a vacuum degasser, a thermostated autosampler, a column oven, a diode array detector and a single-quadrupole mass analyser with electrospray ionization (ESI–MS). Chromatographic separations were carried out at 35 °C on a Kinetex C18 2.6 µm column (100 mm × 3 mm i.d., Phenomenex) in series with a C18 guard column (4 mm × 3 mm i.d.) using 10 µL injections. Eluent A was 5% aqueous acetonitrile with 0.05% formic acid and eluent B was acetonitrile with 0.05% formic acid. The flow rate was 0.8 mL/min and the gradient was as follows: 0–2 min: 10.5–21.1% B; 2–15 min: 21.1–26.3% B; 15–24 min: 26.3–47.4% B; 24–25 min: 47.4–100% B; 25–30 min: 100% B; 30–31 min: 100–10.5% B; 31–35 min: 10.5% B. ESI conditions were as follows: desolvation line (DL) temperature: 250 °C; heat block temperature: 400 °C; drying N_2_ gas flow: 15 L/min; nebulizer N_2_ gas flow: 1.5 L/min; positive ionization mode. Data were acquired and processed using the LabSolutions 5.42v software (Shimadzu).

### Statistical analyses

We analysed the data on the two species and life stages separately. We excluded two individuals due to extremely slow development (development stage was lower than the mean by more than 3 SD), and accidentally lost three individuals. This resulted in the following sample sizes used in the analyses: *R. dalmatina*; control: 35, low *Bd* dose: 36, high *Bd* dose: 35; *B. bufo*: control: 34, low *Bd* dose: 36, high *Bd* dose: 35.

We averaged GE values obtained from qPCR runs for each sample and subsequently rank-transformed means, because GE values that fall outside the standard interpolation curve are not estimated reliably.

In *R. dalmatina* we compared Brevinin-1 Da quantity among treatments using general linear models (GLM) entering log-transformed values of the chromatogram areas as the dependent variable. We used log-transformation to enhance normality of model residuals and homogeneity of variances. In case of 14 days old froglets, we only had 5–5 replicates in *Bd*-exposed treatments because of mortality. For each triplet, we calculated mean values of body mass, development stage and infection intensity. The initial model included treatment as a fixed factor, and body mass, development stage and infection intensity as covariates.

To calculate the number of bufadienolide compounds (NBC) present in each *B. bufo* individual, we assumed a compound to be present if the signal-to-noise ratio (S/N) of its peak was at least three. We estimated the quantity of each compound from the area values of chromatogram peaks based on the calibration curve of the bufotalin standard, and summed up these values to obtain an estimate of total bufadienolide quantity (TBQ) for each individual (for a similar approach see [34, 94]). We analysed effects of treatment on both toxin variables separated by life stages using GLMs. In case of TBQ, we entered as the dependent variable log-transformed values in case of tadpoles and square-root transformed values in case of metamorphs to enhance normality of model residuals and homogeneity of variances. Initial models included treatment as a fixed factor and dry mass, development stage and infection intensity as covariates. In case of a significant treatment effect, we used Tukey HSD post-hoc tests to reveal significant differences between treatment groups. When a covariate also had a significant effect, we ran post-hoc tests on residuals extracted from the regression of the dependent variable on the covariate.

We analysed body mass data using GLMs. Initial models included treatment as a fixed factor and an estimate of the speed of development (Gosner stage in case of tadpoles and length of larval development in case of metamorphs) as a covariate, as well as their interaction. To enhance normality of model residuals and homogeneity of variances, we entered log-transformed values of body mass in case of *R. dalmatina* tadpoles and *B. bufo* metamorphs. In case of the high *Bd* dose treatment in *B. bufo* tadpoles, and both *Bd* treatments in metamorphs, where *Bd* prevalence was high, we also investigated the effect of infection intensity on body mass in the *Bd*-exposed treatment groups with GLMs, including infection intensity and development stage as covariates. We analysed variation in development stage using GLMs with treatment as a fixed factor, except for *R. dalmatina* metamorphs where we used Kruskal–Wallis tests due to the non-normal distribution of model residuals and inhomogeneity of variances. In case of *B. bufo* tadpoles in the high *Bd* dose treatment we analysed the relationship between infection intensity and development stage using Spearman rank correlation and Pearson correlations in both *Bd* treatments of metamorphs.

We verified normal distribution of model residuals using Shapiro–Wilk tests and by inspecting diagnostic plots, and homogeneity of variances using Levene’s tests. We applied a backward stepwise model simplification procedure [95] to avoid potential problems due to the inclusion of non-significant terms [96]. We obtained statistics for removed variables by re-entering them one by one to the final model. All tests were two-tailed. Statistics were calculated using SPSS Statistics 20.0 for Windows.

## Data Availability

All data used in the analyses will be available from Figshare Repository. https://doi.org/10.6084/m9.figshare.12098115 (Ujszegi et al., 2021).

## Abbreviations

AMP: Antimicrobial peptide
Bd: *Batrachochytrium dendrobatidis*
Bsal: *Batrachochytrium salamandrivorans*
GE: Genomic equivalent
GLM: General linear model
GPL: Global pandemic lineage
HPLC: High performance liquid chromatography
MS: Mass spectrometry
NBC: Number of bufadienolide compounds
NE: Norepinephrine
qPCR: Quantitative polymerase chain reaction
RSW: Reconstituted soft water
SD: Standard deviation
TBQ: Total bufadienolide quantity
TFA: Trifluoracetic acid
